# Longer Ubiquinone Side Chains Contribute to Enhanced Farnesol Resistance in Yeasts

**DOI:** 10.3390/microorganisms8111641

**Published:** 2020-10-23

**Authors:** Ruvini U. Pathirana, Cory Boone, Kenneth W. Nickerson

**Affiliations:** School of Biological Sciences, University of Nebraska-Lincoln, Lincoln, NE 68588-0666, USA; pathiranar@uthscsa.edu (R.U.P.); Boone.Cory@huskers.unl.edu (C.B.)

**Keywords:** farnesol resistance, ubiquinone side chain, yeasts, oxidative stress

## Abstract

Ubiquinones (UQ) are intrinsic lipid components of many membranes. Besides their role in electron-transfer reactions there is evidence for them acting as free radical scavengers, yet their other roles in biological systems have received little study. The dimorphic fungal pathogen *Candida albicans* secretes farnesol as both a virulence factor and a quorum-sensing molecule. Thus, we were intrigued by the presence of UQ9 isoprenologue in farnesol-producing *Candida* species while other members of this genera harbor UQ7 as their major electron carrier. We examined the effect of UQ side chain length in *Saccharomyces cerevisiae* and *C. albicans* with a view towards identifying the mechanisms by which *C. albicans* protects itself from the high levels of farnesol it secretes, levels that are toxic to many other fungi including *S. cerevisiae*. In this study, we identify UQ9 as the major UQ isoprenoid in *C. albicans,* regardless of growth conditions or cell morphology. A *S. cerevisiae* model yeast engineered to make UQ9 instead of UQ6 was 4–5 times more resistant to exogenous farnesol than the parent yeast and this resistance was accompanied by greatly reduced reactive oxygen species (ROS) production. The resistance provided by UQ9 is specific for farnesol in that it does not increase resistance to high salt (1M NaCl) or other oxidants (5 mM H_2_O_2_ or 1 mM menadione). Additionally, the protection provided by UQ9 appears to be structural rather than transcriptional; UQ9 does not alter key transcriptional responses to farnesol stress. Here, we propose a model in which the longer UQ side chains are more firmly embedded in the mitochondrial membrane making them harder to pry out, so that in the presence of farnesol they remain functional without producing excess ROS. *C. albicans* and *Candida dubliniensis* evolved to use UQ9 rather than UQ7 as in other *Candida* species or UQ6 as in *S. cerevisiae*. This adaptive mechanism highlights the significance of UQ side chains in farnesol production and resistance quite apart from being an electron carrier in the respiratory chain.

## 1. Introduction

*Candida albicans*, a member of normal human flora has become the most common nosocomial fungal pathogen in humans [1]. This yeast shows remarkable diversity in its morphology and secretes farnesol, a quorum-sensing molecule which mediates yeast to hyphae dimorphism in a cell density-dependent manner without influencing growth rate [2]. Farnesol is a 15-carbon sesquiterpene which is formed from farnesyl pyrophosphate, an intermediate in the ergosterol biosynthetic pathway [3]. In vivo, this lipophilic molecule acts as a virulence factor which enhances systemic candidiasis in mice [4] and stimulates immune recognition by host macrophages [5]. Farnesol stimulated an 8.5-fold increase in macrophage migration in vitro and a 3-fold increase of peritoneal infiltration by mouse macrophages in vivo [5]. Wild type *C. albicans* tolerates up to 300–500 µM farnesol [3,6] while farnesol production can vary from 0.5 µM to 50 µM, depending on strain differences as well as planktonic or biofilm growth [2,7,8,9]. The ability of *C. albicans* to tolerate such high concentrations of farnesol is remarkable because much lower concentrations of farnesol (<20–25 µM) are antimicrobial towards other microorganisms including both bacteria and fungi. The growth of *S. cerevisiae*, a distant cousin of *C. albicans*, is inhibited by <25 µM farnesol which causes cell cycle arrest and/or mitochondrial dysfunction [10,11]. Farnesol can also induce apoptotic cell death in filamentous fungi such as *Aspergillus nidulans* and *Aspergillus fumigatus* [12] as well as *Fusarium graminearum* [13]. Farnesol also induces reactive oxygen inside macrophages leading to increased intracellular oxidative stress [14]. The juxtaposition of these observations poses the question of the mechanisms whereby *C. albicans* tolerates farnesol doses that are lethal to other fungi; what adaptations has it made to be able to use farnesol as a quorum-sensing molecule and a virulence factor?

Current best evidence is that farnesol kills cells by generating superoxide radicals, the major reactive oxygen species (ROS), by interacting with the mitochondrial electron transport chain during respiratory growth of *S. cerevisiae* [10,12,15]. ROS production is a normal byproduct of respiration and mitochondria have their own antioxidant mechanisms for the efficient elimination of superoxide anions [16] which can be localized either in the mitochondrial matrix, inner membrane/outer side, outer membrane/inner side, or intermembrane space [16]. Presumably farnesol enhances ROS production to a level such that the cell can no longer cope. 

Thus, it is reasonable to speculate that a cell or organism could increase its farnesol tolerance by structural adaptations to mitochondrial membranes or other components in order to avoid leakage of electrons, thus minimizing cell damage and/or reduced proton motive force. 

**Figure 1:**
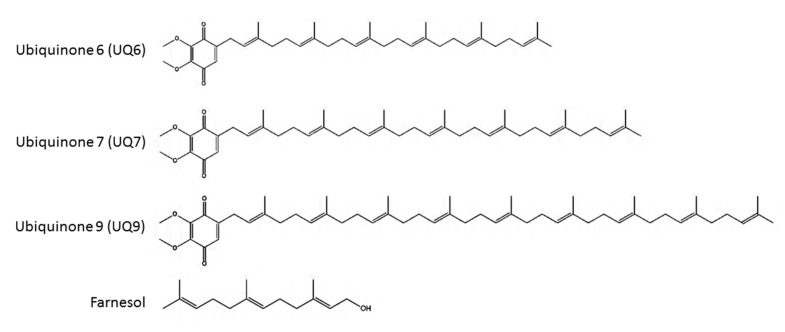
Structures of ubiquinones and farnesol. Ubiquinone consists of a benzoquinone ring (aromatic moiety) and a side chain of variable isoprenoid units. *S. cerevisiae* has six isoprenoid units in its side chain (UQ6), while most *Candida* sp. have seven (UQ7), and *C. albicans* has nine (UQ9). Farnesol, is a C_15_ sesquiterpene alcohol with only three isoprenoid units.

One of the major sites responsible for electron leakage in the mitochondrial electron transport chain is ubiquinone, especially when electrons translocate to complex III via reduction of ubiquinone to ubiquinol [15,16]. Ubiquinones (UQ) are critical membrane-localized electron carriers, which ‘ubiquitously’ exist in the mitochondrial electron transport chain in all living organisms. They are involved in the transfer of electrons from NADH-ubiquinone oxidoreductase (complex I) and succinate-ubiquinone reductase (complex II) to ubiquinol-cytochrome *c* oxidoreductase (complex III) [15,16]. They have a redox active benzoquinone ring attached to a lipophilic side chain consisting of a variable number of isoprene units (Figure 1). The number of isoprenoid units varies among species, ranging from 4 to 14, where the number of isoprenoid units is numerically designated following the abbreviation UQ or Q for ubiquinone. The hydrophobic isoprene tail is derived from successive head-to-tail condensations of isoprene units [isoprenyl diphosphate (IPP)] catalyzed by prenyl diphosphate synthase [17]. The specificity of this enzyme determines the number of isoprenoid units present (i.e., side chain length) and, therefore, it is believed that the isoform of this enzyme varies among different species [17,18]. Polyprenyl diphosphate synthase is encoded by a single gene *COQ1* in both *S. cerevisiae* [19] and *C. albicans* [20] while its homolog in *Arabidopsis thaliana* is identified as *At2g34630* [21]. 

The variability of ubiquinone side chain length is often taxonomically useful [22] but functional differences due to differing UQ side chain lengths have rarely been reported. Thus, we were intrigued by taxonomic studies which showed that *S. cerevisiae* produces UQ6 while most *Candida* species produce UQ7 except for *C. albicans* and *C. dubliniensis* which have UQ9 [23,24]. Significantly, *C. albicans* and *C. dubliniensis* are major human fungal pathogens which cause life-threatening systemic infections in humans and they are the only two species in the *Candida* clade known to excrete large quantities of farnesol [8,9]. As would be expected for organisms using farnesol in this fashion, the growth rates of *C. albicans* and *C. dubliniensis* were not affected by 300 µM [2,6] and 150–200 µM [25,26] farnesol, respectively. These observations led us to hypothesize that a longer isoprenoid chain length may provide an advantage to farnesol-producing *Candida* species, allowing them to cope with the oxidative stress exerted by the ROS generated by farnesol. In this study, we tested this hypothesis by comparing the farnesol sensitivity of yeasts, using a recombinant *S. cerevisiae* which produces UQ9 instead of UQ6 [21]. Our findings suggest that a longer isoprenoid side chain length protects yeasts from farnesol and excess ROS by a mechanism independent of transcriptional regulation. Thus, synthesizing UQ9 instead of UQ7 could serve as a pathogen-adapted structural trait in *C. albicans*. 

## 2. Materials and Method

### 2.1. Yeast Strains and Media

The yeast strains used in this study are listed in Table 1. *C. albicans* SC5314 and WO-1 were obtained from Alexander Johnson, University of California at San Francisco, and David Soll, University of Iowa, respectively. *S. cerevisiae* BY4741 and its isogenic mutants, *Δcoq1*::pYES, *Δcoq1*::pYES + *At2g34630*, and *Δcoq1*::pYES + *COQ1* were obtained from Gilles Basset, University of Nebraska-Lincoln [21]. The *Δcoq1*::pYES was effectively a petite strain in that it was unable to grow on glycerol + ethanol agar plates [21]. The expression capacity of the pYES system was ca. 60% of the BY4741 parent, with pYES + COQ1 producing UQ ≤6 and pYES + At2g34630 producing UQ 7–9 [21].

Yeast extract-peptone-dextrose (YPD) medium containing 1% yeast extract, 1% peptone, and 2% glucose was used for routine growth of yeast stock cultures and in farnesol sensitivity assays. YPD was supplemented with 0.05% (*w*/*v*) D-galactose as an inducer for all *S. cerevisiae* strains, and G418 (Geneticin purchased from Gold Biotechnonlgy, St. Louis, MO, USA, 200 µg/mL) as the selective antibiotic for strains constituted with plasmid vector unless specifically mentioned. The glucose-salts-biotin (GSB) medium contained 1 g/L of (NH_4_)_2_SO_4_, 2 g/L of KH_2_PO_4_, 50 mg/L of MgSO_4_.7H_2_O, 50 mg/L of CaCl_2_.2H_2_O supplemented with sterile 30 mL of a 50% (wt/vol) glucose stock and 0.4 mL of the vitamin stock (containing 0.002% biotin, 0.02% pyridoxine and 0.02% thiamine in 20% ethanol) [2]. GSB was used as a minimal medium for preparing *C. albicans* cell stocks for inoculum preparation and ubiquinone analysis. Modified glucose-proline-phosphate (GPP) medium [27] was used for preparation of anaerobic cells from *C. albicans* SC5314. Synthetic complete (SC) medium used to grow opaque cells from *C. albicans* SC5314 was composed with 0.67% yeast nitrogen base, 2% glucose and supplemented with appropriate amino acids [28]. 

### 2.2. Inoculum Preparation

Inocula were grown from single colonies of *C. albicans* SC5314 and WO-1 in YPD at 30 °C for 16–18 h with shaking at 225 rpm. The stationary phase cells were harvested by centrifugation at 5000 rpm in Beckman J-21C centrifuge and washed three times in 50 mM potassium phosphate buffer (pH 6.5). These cells were inoculated into minimal media (GSB) and grown at 30 °C in a shaking incubator at 225 rpm until they reached a stationary phase whereupon they were harvested and washed as described above and then stored at 4 °C. The cells were used within a week of preparation. The *C. albicans* WO-1 opaque cells were grown in SC medium to stationary phase whereupon the cells were harvested and stored at 4 °C as described above. 

### 2.3. Ubiquinone Analysis in C. albicans

To analyze ubiquinone content in *C. albicans*, the cells were grown in 40 mL of either rich (YPD) or minimal media (GSB, GPP with N-acetylglucosamine, or SC) at 30 or 37 °C for 18–24 h as appropriate to induce the different morphological stages of *C. albicans*, i.e., yeasts or mycelia, white or opaque cells, or anaerobically grown cells. The morphology of each cell type was confirmed by microscopy before harvesting. Cells were harvested by centrifugation at mid-exponential phase, washed once with 25 mL of water, and resuspended in 1 mL of water whereupon cell number was quantified by absorbance at 600 nm and the cell numbers normalized by adjusting the volume of each sample. One ml of each suspension was transferred to a 10mL pyrex tube containing 0.5 mL of 0.5 mm glass beads, spiked with 8.5 nmoles of UQ-10, and vortexed for 90 s. Samples were then mixed with 2 mL of 95% ethanol, heated at 70 °C for 10 min, cooled and extracted twice with 5 mL hexane. The hexane layers were combined, evaporated to dryness with N_2_, and resuspended in 1 mL of methanol: dichloromethane (10:1). The types of UQ present in the ubiquinone pool were identified as described by Ducluzeau et al. [21] using high-performance liquid chromatography (HPLC) in diode array detection (DAD) mode with UQ10 as the internal standard [21]. Extracts from cell suspensions were analyzed at 30 °C by HPLC (Agilent, Santa Clara, CA, USA) on a 5 µM Supelco Discovery C-18 column (250 × 4.6 mm, Sigma-Aldrich, St. Louis, MO, USA) in isocratic mode at a flow rate of 1 mL min^−1^ with methanol:hexane (95:5 *v*/*v*) for spectrophotometric detection of quinones at 275 nm. Quinol species had been fully reoxidized during heating and were quantified as a part of the quinone pool. Retention times for the ubiquinones were 8.6 min for UQ-6, 11.5 min for UQ-7, 15.8 min for UQ-8, 22.1 min for UQ-9, and 31.5 min for UQ-10. The typical recovery value for yeast ubiquinones is approximately 90% and data were corrected accordingly. All glassware used for culture growth and analysis were washed with 2M NaOH and then rinsed with Milli-Q water followed by a final rinse in acetone.

### 2.4. Farnesol Sensitivity Assays—High Aeration

Stock cells were prepared as described above and used at an initial OD_600_ = 0.02 (6 × 10^5^ cells/mL). These cells were tested for their sensitivity to farnesol via a series of growth curves and live dead cell assays at different farnesol concentrations (0, 50, and 100 µM farnesol in methanol) where the final methanol concentration never exceeded 1%, a concentration that had no effect on cell growth or death [2]. Growth studies were undertaken with 50 mL YPD or GSB in 250 mL glass Erlenmeyer flasks with rotary agitation at 225 rpm, measuring optical density at 600 nm in a Spectra MaxPlus Microplate Spectrophotometer. Cell death was followed by staining the yeast cultures with 0.05% methylene blue at specified time points. For all experiments, farnesol was present from time zero and the 0 farnesol samples still contained methanol, thus serving as methanol-only controls which did not inhibit growth or promote cell death.

### 2.5. Farnesol Sensitivity Assays—Low Aeration

Fresh single colony isolates were grown overnight at 30 °C in 4 mL of YPD supplemented with G418 (200 µg/mL) for selection and D-galactose (0.05% *w*/*v*) for induction. Cultures were tested for their farnesol sensitivity in sterile Corning costar 96-well flat bottom plates with low evaporation lids. The overnight cultures were diluted to OD_600_ = 0.1 with YPD + D-galactose. Each well contained 200 µL of culture and farnesol (0, 50, 100, 200, or 400 µM) in four replicates. The farnesol stock solution was 400 mM in methanol so that after dilution each culture had ≤0.1% methanol. Growth assays were performed at 30 °C in a Biotek Synergy H1 Hybrid Reader using Gen5 Microplate reader software v2.0.01.14. Plates were oscillated for one minute every 10 min before the OD_600_ was recorded. The growth data was exported as Microsoft excel spreadsheet files and assessed with Sigma Plot 12.

### 2.6. Measurement of Intracellular Reactive Oxygen Species (ROS) via DHR123 Staining

Synchronized yeast cells were obtained by diluting an overnight culture of stationary phase cells in YPD liquid media to 0.1 OD_600_ and incubating it at 30 °C with shaking until it reached 0.5 OD_600_. These mid-log cells were subdivided into flasks and then treated with 0–400 µM farnesol or 10 mM hydrogen peroxide as the positive control. After 4 h shaking, 1 mL samples from each flask were dispersed into 5 mL polystyrene round bottom tubes (BD Biosciences No. 352058) and immediately stained for ROS with 1 µg/mL Dihydrorhodamine 123 (DHR123, Sigma Aldrich D1054, St. Louis, MO, USA) in dimethylsulfoxide and incubated at 37 °C for 30 min in the dark. The fluorescent intensity of the cells was analyzed immediately by a BD^®^ FACSCantor flow cytometer using triplicate samples. Data are presented as the fold change, i.e., the ratio of the fluorescence/basal fluorescence with no farnesol or H_2_O_2_. In this procedure, the freely permeable, non-fluorescent DHR123 enters the cells where it is oxidized by ROS in the cytoplasm, resulting in formation of the fluorescent dye which is then localized in the mitochondria [31].

### 2.7. Measurement of Oxygen Consumption Rate in Yeasts

Cells (25 mL) were grown aerobically to mid logarithmic phase with rotary agitation at 225 rpm, harvested, washed twice in YEP medium [1% yeast extract, 1% peptone and 0.5% sodium chloride (pH 7.0)] and resuspended in 700 µL of the same medium. Glucose was added to each sample to a final concentration of 2% right before analysis and oxygen consumption was monitored using a Clark-type oxygen electrode (Rank Brothers, Cambridge, UK) until all oxygen in the sample chamber was consumed. Calibration of the Clark electrode for maximum oxygen consumption was done under normal atmospheric conditions and the slope was calculated for each sample when oxygen consumption was linear. The rate of respiration was normalized to the cell number in the chamber (OD 600 nm) and expressed as % O_2_/min/OD_600_. The values reported were the means ± standard deviation (SD) of three or four replicates.

### 2.8. Salt and Oxidative Stress Assays

The yeast strains to be tested were grown in liquid YPD culture to mid-log phase and then spotted onto YPD + 0.05% galactose agar plates with and without the salt or oxidant being tested, with inocula at a series of 10-fold dilutions. Plates were photographed after 48 h at 30 °C.

### 2.9. Catalase Activity Induced by Sub-Lethal (20 µM) Farnesol

Yeast cells were grown to exponential phase in rich media (YPD) and exposed to sub-lethal concentrations of farnesol (20 µM) for 60 min. The cells were harvested just after farnesol treatment and catalase activity was determined by a colorimetric assay using the EnzyChrom catalase assay kit (BioAssay Systems, Hayward, CA, USA) according to the manufacturer’s instructions. The test was performed in three biological replicates and results were analyzed using the Student *t*-test at *p* < 0.05.

## 3. Results

### 3.1. UQ9 is the Major Ubiquinone in *C. albicans*

**Figure 2:**
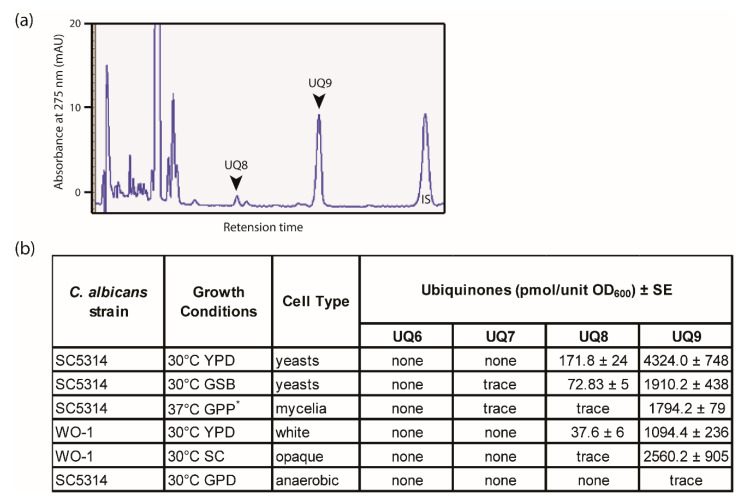
Ubiquinone analysis in physiologically distinct *C. albicans* morphological types. The analysis was undertaken by high-performance liquid chromatography (HPLC) in diode array detection (DAD) mode using UQ10 as the internal standard (IS) [21]. (**a**) Representative histogram from ubiquinone profile of *C. albicans* cells grown in synthetic (glucose-salts-biotin, GSB) medium. (**b**) Quantification of ubiquinones as detected by HPLC-DAD. Trace indicates that the ubiquinone detected was below the sensitivity limit of 0.1 pmol unit^−1^ OD600 nm. * Glucose-proline-phosphate (GPP) medium is supplemented with N-acetylglucosamine as an inducer of hyphal growth. GPD is the defined medium developed by Dumitru et al. [27] for anaerobic growth of *C. albicans*. Data are means ± standard deviation (SD) of duplicate measurements.

The diversity of ubiquinone presence in genus *Candida* was studied in detail by Suzuki and Nakase [23,24] where UQ9 was identified as the major ubiquinone in *C. albicans* strain JCM 1542 (IFO 1385). Ubiquinones are known to be localized in different types of cell membranes. Due to the distinct structural and chemical compositions in different morphologies of *C. albicans*, we analyzed the ubiquinone pool in *C. albicans* cells grown under different physiological conditions and/or in different morphological states, i.e., yeasts, hyphae, anaerobically grown cells, and both white and opaque cells (Figure 2). We confirmed that UQ9 is the major type of UQ in *C. albicans* in two widely used laboratory strains SC5314 and WO-1 (Table 1) as analyzed by HPLC-DAD with UQ10 as the internal standard [21]. Figure 2a shows the retention profile of ubiquinones in wild type *C. albicans* grown in minimal media and Figure 2b summarizes the ubiquinone types and total ubiquinone content under different physiological and morphological states. These data confirm the predominance of UQ9 in all cell types except for the anaerobically grown cells which lack detectable ubiquinones. In particular, UQ9 was the dominant ubiquinone in both rich medium (YPD) and minimal medium (GSB), for both white cells and opaque cells, and for both mycelial cells (37 °C) and yeast cells (30 °C). These observations show that UQ9 is a general characteristic of *C. albicans* regardless of the cell morphology. We did not detect any UQs in anaerobically grown cells; this absence is expected because mitochondrial respiration is absent in anaerobically growing cells.

### 3.2. Farnesol Resistance in *S. cerevisiae* Depends on the UQ Isoprenologue, UQ6 vs. UQ9

**Figure 3:**
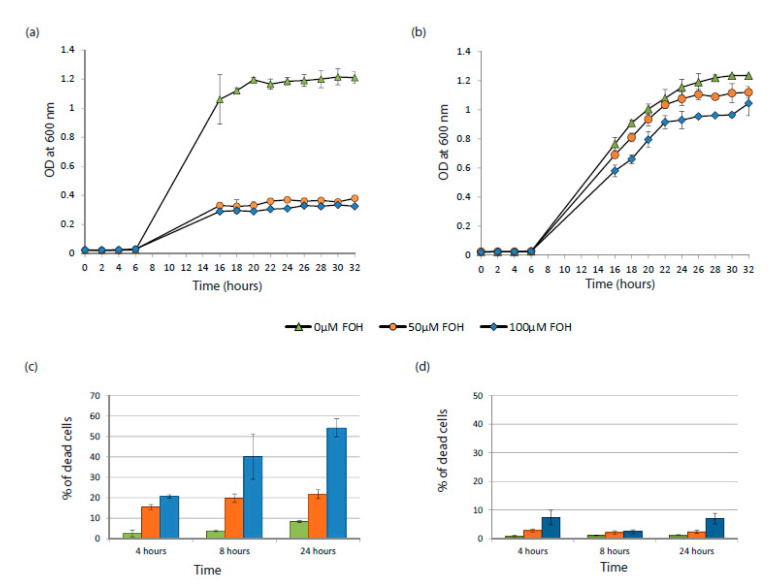
Longer ubiquinones increase the farnesol survival in *S. cerevisiae*—high aeration. Growth curves in yeast extract-peptone-dextrose (YPD) of (**a**) *S. cerevisiae* BY4741 and (**b**) *S. cerevisiae* *Δcoq1* + *At2g*34630 in the absence of farnesol (triangles ▲) and in the presence of 50 µM (spheres ●) or 100 µM (diamonds ◆) farnesol. Effect of farnesol on cell death for (**c**) *S. cerevisiae* BY4741 and (**d**) *S. cerevisiae* Δcoq1 + At2g34630. Cultures were grown in the absence of farnesol (green solid fill) and in the presence of 50 µM (orange solid fill) or 100 µM farnesol (blue solid fill). For each time point, the bars are presented in sequence for 0, 50, and 100 µM farnesol. Cell viability was determined by methylene blue staining as the means ± standard error of triplicate experiments from YPD cultures after 4, 8, and 24 h growth.

**Figure 4:**
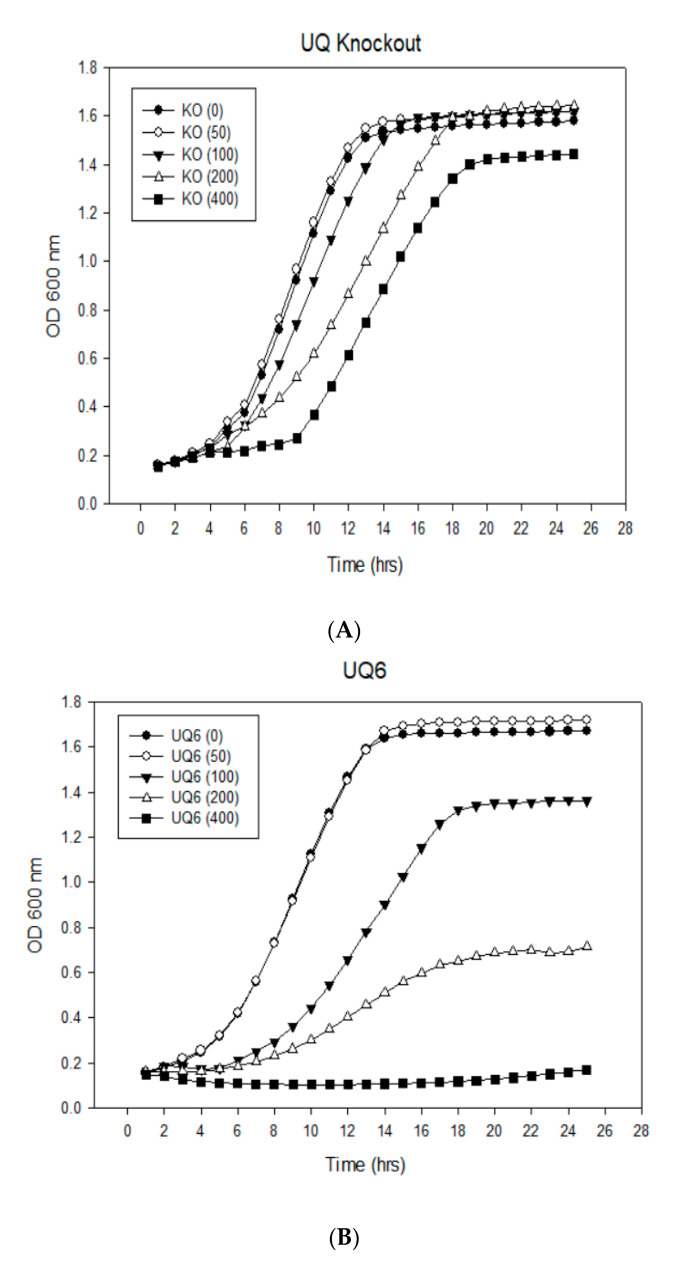

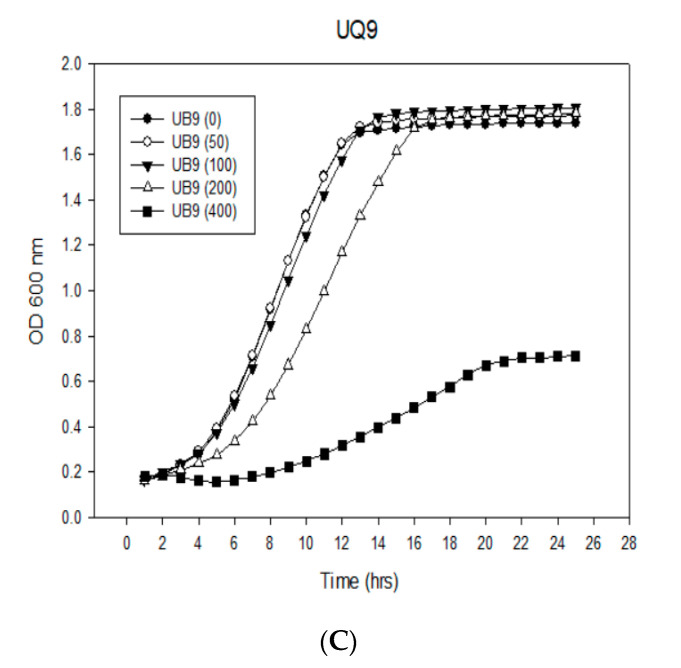
Longer ubiquinones increase the farnesol survival in *S. cerevisiae*—low aeration. (**A**) UQ knockout, *Δcoq1*::pYES. (**B**) UQ6, *Δcoq1*::pYES + coq1. (**C**) UQ9, *Δcoq1*::pYES + At2g34630. All are triplicate growth curves in YPD with D-galactose and 0 (●), 50 (○), 100 (▼), 200 (Δ), or 400 (◼) µM farnesol. The triplicate values were all within +5%.

The importance of ubiquinone chain length for farnesol resistance was tested by comparing strains of *S. cerevisiae* which produce UQ6 (wild type BY4741 and *∆coq1::*pYES + COQ1) and UQ9 (*Δcoq1::*pYES + *At2g34630)* [21]. Growth levels were observed in the presence and absence of 50–400 μM farnesol (Figure 3 and Figure 4). The two sets of experiments differ primarily in the degree of aeration achieved. Figure 3 describes high aeration growth, 50 mL of YPD in 250 mL Erlenmeyer flasks shaking continuously at 225 rpm, whereas Figure 4 describes low aeration growth, 0.2 mL of YPD per well in 96-well plates shaken for one min every 10 min. The 96-well plate format is technically advantageous for measuring the growth of triplicate samples at 10 min intervals over 25 h while using minimal amounts of farnesol. Additionally, the lower aeration shifts cellular metabolism towards a greater reliance on fermentation, allowing us to confirm our expectation that farnesol inhibits respiration [6,10] but not fermentation [27].

Our high aeration results in YPD (Figure 3) showed 3–4 fold increased resistance to farnesol in the UQ9 producing strain compared with its UQ6 producing parent. This increased resistance to 50 and 100 µM farnesol for the UQ9-producing strain was evident in both the higher cell yields achieved (compare Figure 3b vs. Figure 3a) and the lower percentage of dead cells observed (compare Figure 3d vs. Figure 3c). Very similar results were obtained when the two yeast strains were grown in three defined, minimal growth media, namely glucose-salts-biotin (GSB), yeast nitrogen base (YNB), and glucose-phosphate-proline (GPP). In each case the BY4741 parent (UQ6) grew poorly in 50 µM farnesol and poorly (GSB) or not at all (YNB and GPP) in 100 µM farnesol whereas the UQ9-producing strain grew equally as well with and without 50 µM farnesol and 50–70% as well with 100 µM farnesol (data not shown).

Similar results were observed for experiments carried out in 96-well plates under low aeration except that the yeast cells were less sensitive to farnesol. In this case the UQ6 yeasts were inhibited only slightly by 50 µM farnesol (Figure 4B) and the cell yield achieved by UQ6 yeasts in 100 µM farnesol (Figure 4B) was 60% of the UQ9 yield (Figure 4C). These results are consistent with the low aeration conditions shifting cellular metabolism from respiration to fermentation. They reinforce the view that farnesol itself is not toxic to yeast cells, but instead it is the ROS generated by farnesol’s interaction with respiring mitochondria which is toxic. Taken together these results agree with those of Okada et al. [32] that under most growth conditions the length of the UQ tail makes very little difference but in the presence of farnesol (Figure 3 and Figure 4) it makes a substantial difference.

### 3.3. Intracellular Reactive Oxygen Species (ROS) Accumulation Decreased with UQ9

**Figure 5:**
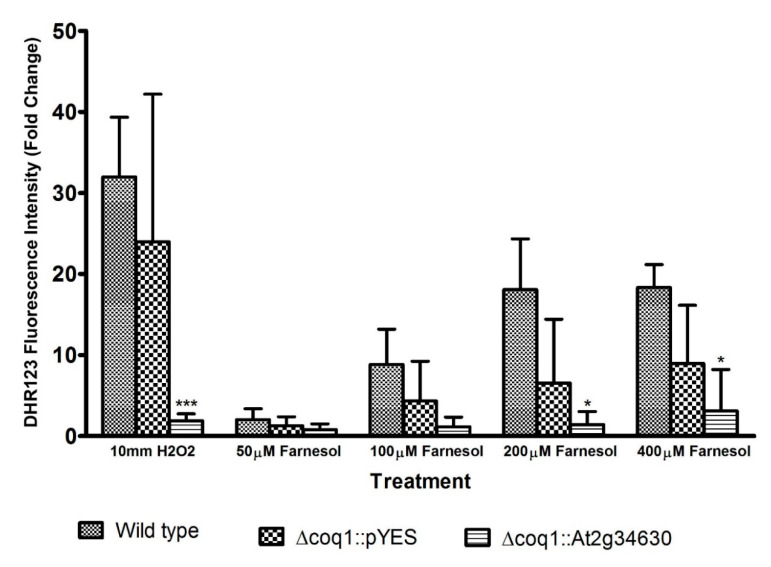
Reactive oxygen species (ROS) accumulation decreases with longer isoprenoid chain length. Flow cytometric analysis of ROS accumulation using DHR123 fluorescence intensity, expressed as the fold change (fluorescence/basal fluorescence with no farnesol or H_2_O_2_). The data represent the mean ± standard deviation for three independent experiments. * = *p* < 0.05; *** = *p* < 0.001.

Farnesol promotes generation of ROS, which subsequently inhibits growth in yeasts [10]. We examined the effect of longer isoprenoid chains on ROS production induced by farnesol. Intracellular ROS was measured via oxidation of the ROS-specific dye DHR123 to fluorescent rhodamine123. As shown in Figure 5, *S. cerevisiae* produces less ROS with UQ9 as the major electron carrier than with UQ6, in a dose-dependent manner increasing from 0 to 200 or 400 μM farnesol. Farnesol concentrations of 200 and 400 µM are both well above the minimal inhibitory concentration value for wild type *S. cerevisiae* and thus the similar fold increases observed for BY4741 (Figure 5) likely reflect the maximum capacity for mostly dead cells. These experiments used H_2_O_2_ (10 mM) as the positive control and, at this lethal concentration, the UQ9 containing strain (*Δcoq1*::pYES + *At2g34630*) also produced significantly less ROS than the wild type. This observation could be due to a more rapid protective response to H_2_O_2_ in the presence of the more firmly embedded UQ or to the impact of a still to be discovered protective mechanism whereby UQ9 prevents or retards cell death in the presence of an otherwise lethal concentration of H_2_O_2._ Our findings indicate that intracellular ROS accumulation decreased with the presence of a longer isoprenoid chain in UQ.

### 3.4. Respiratory Rate Remains Unchanged Regardless of UQ Isoprenologue

The replacement of UQ6 with UQ9 leads to greater farnesol resistance and less intracellular ROS. These changes could be due to ubiquinone’s structural role in mitochondria or they could be a secondary consequence of the UQ9-containing cells having less efficient respiration or a shift in metabolism towards more fermentation and less respiration. Therefore, we compared *S. cerevisiae* BY4741 and *Δcoq1*::pYES + *At2g34630* and found that their respiration rates were 4.27 ± 0.30 and 4.02 ± 1.29% O_2_/min/OD_600_, with UQ6 and UQ9, respectively. This equivalence in mitochondrial function suggests that the farnesol resistance observed is not due to slow or differential respiratory rates. These results were expected because Okada et al. [32] found that even though *S. cerevisiae* prefers UQ6, it could grow well with all UQ chain lengths between 5 and 10. In SC minimal medium containing glycerol, the maximum growth rates with UQ8 and UQ9 were only slightly less than with UQ6 [32].

### 3.5. Oxidative Sensitivity and Osmotolerance in UQ6 vs. UQ9

**Figure 6:**
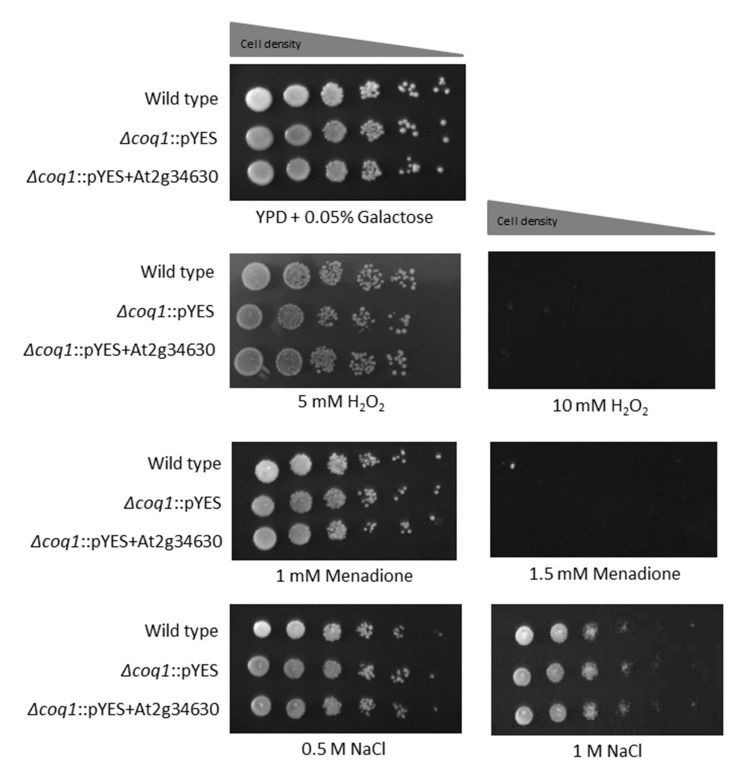
Sensitivities to other stress conditions. Serial 10-fold dilutions of cells were spotted onto YPD +0.05% galactose agar plates containing the indicated salt or oxidant and photographed after 48 h at 30 °C.

We also wished to distinguish whether the enhanced resistance to farnesol exhibited by the UQ9 containing yeasts is due to the UQ9 itself or to a secondary physiological change triggered by the UQ9. Thus, we compared the yeasts for their oxidative and osmotolerance towards common stress agents. We observed the growth effects on YPD agar in the presence of added H_2_O_2_, menadione, and sodium chloride (Figure 6). *Δcoq1::*pYES is effectively a petite and as expected it did not grow on glycerol/lactate plates (see Figure 4A of [21]). The wild type BY4741 and recombinant *S. cerevisiae Δcoq1::*pYES + *At2g34630* use UQ6 and UQ9, respectively. We did not observe any differential responses to these stress agents based on their ubiquinone contents (Figure 6). A separate set of experiments with and without the selective agent G418 showed virtually identical growth patterns, thus indicating that plasmid loss was not a problem in this timeframe. We conclude that general oxidative tolerance and osmotolerance are unaffected by ubiquinone type. This conclusion is consistent with reports that UQ deficient mutants tolerate most types of oxidative stress [33].

### 3.6. Catalase Assays

**Figure 7:**
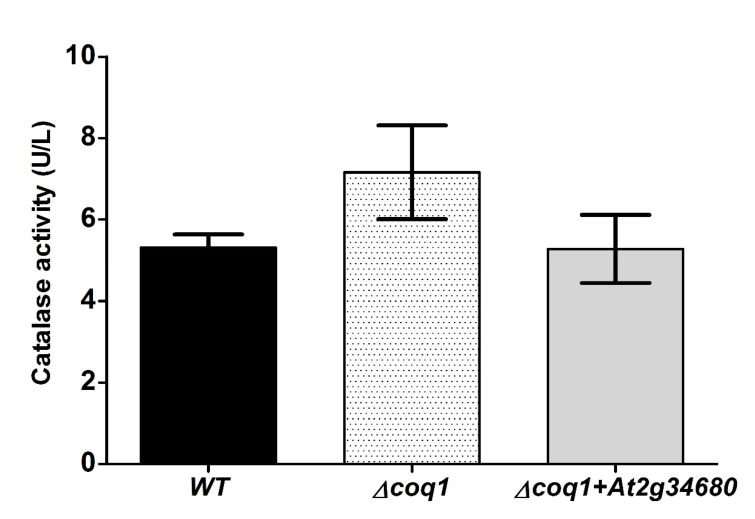
Change in UQ side chain length does not alter transcriptional regulation of the oxidative stress response. Catalase activity (units/liter) measured 60 min after addition of sub-lethal farnesol (20 µM). Average of 3 replicates.

Catalase (Cat1p) is a key enzyme in protecting cells from oxidative stress. It detoxifies hydrogen peroxide and other oxidants, thereby restoring redox homeostasis. Farnesol (50 μM) induces dose-dependent increases in *CAT1* expression in *C. albicans* [24] and therefore catalase activity should be induced by farnesol stress. Accordingly, we compared the catalase activities in UQ9- and UQ6- containing yeasts but we did not find any significant differences in basal catalase activity or catalase induction capacity (Figure 7). We conclude that Cat1p activity is unaffected by ubiquinone type.

## 4. Discussion

The fungal pathogen *C. albicans* exhibits a remarkably greater ability to withstand oxidative stress than do the model yeasts *S. cerevisiae* and Schizosaccharomyces pombe [34,35]. The distinctive and robust stress tolerance mechanisms in *C. albicans* coevolved with host niche-specificity in order to evade the oxidative killing pressures exerted by hostile immune protective mechanisms [35,36,37]. However, the greater stress tolerance of *C. albicans* cannot be attributed solely to transcriptional regulation of stress responsive genes since the antioxidant genes (CAT1, GPX, SOD) and the ROS scavenging systems (glutathione/glutaredoxin, thioredoxin) are equally upregulated in all yeasts [37,38,39]. Thus, the remarkable tolerance to farnesol to *C. albicans* compared with *S. cerevisiae* suggests the evolution of specific structural adaptations as well as the general antioxidant responses just described. Here we have shown that the structural differences inherent in having membrane bound UQ9 rather than UQ6 provides a significant advantage in tolerating farnesol and the oxidative stress induced by farnesol. The added protection provided by UQ9 appears to be specific for farnesol and due to the UQ9 itself, i.e., it is not a secondary effect. These conclusions are based on the fact that the UQ6 and UQ9 cells had equivalent osmotolerance (to 1 M NaCl) and redox tolerance (to 5 mM H_2_O_2_ and 1 mM menadione) (Figure 6). They also had equivalent levels of catalase activity (Figure 7) and preliminary analysis showed that they had equivalent amounts of total and phosphorylated Hog1p both before and after stress with 10 mM H_2_O_2_ or 50–100 µM farnesol (data not shown). These findings support our suggestion that the UQ9 side chains have evolved as a structural adaptation to farnesol rather than as a superior general stress mechanism. According to our findings, it is very unlikely that transcriptional regulation of oxidative protection is affected by the type of endogenous UQ present. Also identifying the contribution of UQ9 side chains to high farnesol resistance in *C. albicans* points to polyprenyl diphosphate synthase (Coq1p) as a potential target to control the dimorphic pathogenicity of this fungus.

Farnesol’s interaction with the mitochondrial membrane is likely responsible for the increased toxicity (Figure 3) and excess ROS production (Figure 5) associated with UQ6 yeasts. This connection between farnesol, ROS, and mitochondrial respiration is consistent with prior reports that some petite mutants of *S. cerevisiae* are farnesol resistant [10,15] while anaerobically grown *C. albicans* did not produce farnesol and did not respond to exogenous farnesol at concentrations ranging from 0 to 1.2 mM [27]. Note that 1.2 mM farnesol represents the maximum solubility of farnesol in water [27]. The enhanced resistance to farnesol was also observed for *S. cerevisiae Δcoq1*::pYES strains (Figure 5). This strain lacks a complete aerobic respiration and is unable to grow in media containing only non-fermentable carbon sources such as glycerol, thus is considered as a petite.

Machida et al. [10] showed that for *S. cerevisiae* farnesol generated reactive oxygen species (ROS) by interacting with the mitochondrial electron transport chain. Here we corroborate their findings while providing the added suggestion that UQ release or solvent exposure is one of the mechanisms for ROS formation. Okada et al. [32] produced mutants of *S. cerevisiae* which synthesized UQ with chain lengths varying from UQ5 up to UQ10. These mutants had roughly equivalent growth rates and respiration rates [32]. Although *S. cerevisiae* has frequently been used as a model for *C. albicans*, it is primarily non-pathogenic and it lacks the diverse morphogenic forms as in *C. albicans*. Because *C. albicans* uses farnesol as a virulence factor in pathogenesis [4,5], it would be interesting to make similar mutants of *C. albicans*, forced to use isoprenoid chain lengths varying from UQ5 to UQ10, and then compare their respective levels of pathogenicity in mouse models of disseminated candidiasis. However, the construction of respiratory deficient mutants in *C. albicans* could be challenging because respiratory deficiency is usually lethal in almost all species of Candida [40].

The wide distribution of UQs in membranes of Golgi, lysosomes, peroxisomes, and cell membranes other than mitochondria suggests UQ may be involved in roles other than electron transfer as reviewed by [41,42,43]. The antioxidant function of UQ is well studied in that it protects against lipid peroxidation in the membrane [44]. All these mechanisms are attributable to the redox active benzoquinone ring of UQ. However, the biological significance of the ubiquinone side chain length is reported only intermittently and no molecular or signaling mechanisms have been suggested for those observations. Okada et al. [32] proposed that the UQ chain length was determined by the membrane hydrophobicity of an organism while Katsikas and Quinn [45] suggested that UQ chain length aids in the localization of UQ within phospholipid bilayers with longer chain lengths permitting efficient transmembrane movement of hydrogen atoms [45].

Most interestingly, *E. coli* was recently found to accumulate far greater levels of its native UQ8 (110-fold) in response to osmotic stress (750 mM NaCl) [46]. The extra UQ8 increased membrane stability quite independent of its role in respiration or radical scavenging. Sévin and Sauer [46] concluded that the osmoprotection provided by UQ8 was conveyed by the octaprenyl tail and not by the benzoquinone moiety, and that only chains of sufficient length were able to provide osmotolerance. They also suggested that UQ_n_ with n > 8 isoprenoid units could provide mechanical stabilization of cellular membranes but no further mechanisms for this osmoprotection have yet been identified. Due to the variety of environmental niches where *E. coli* can be found [47], this physical mechanism could be another evolutionary adapted trait in these bacteria to increase their fitness in both host-associated and non-host associated habitats [47]. With our observation of farnesol protection conferred by longer UQ side chain length, we conclude that having different isoprenoid side chain lengths in different organisms is another example of niche-adapted evolutionary mechanisms by which chemical diversity is generated in nature in order to provide a selective advantage. The importance of farnesol as a virulence factor for *C. albicans* is such that using UQ9 is just one of the structural adaptations this successful pathogen has made. Another possible adaptation is the pressure farnesol provides for maintaining *C. albicans* as a diploid rather than as a haploid [48]. In addition to acting as a virulence factor [4,5] and signaling molecule [2,3], farnesol has some detergent-like activity. Another example of the complexity of cellular adaptations to detergents has been studied in enteric bacteria growing in the presence of 5–10% sodium dodecyl sulfate (SDS). For *Enterobacter cloacae* and *E. coli*, their structural adaptations to SDS occur in at least five subcellular locations [49,50]. Moving progressively inwards, these were (i) a negatively charged capsule provided by the presence of colanic acid [49]; (ii) the outer membrane as a necessary barrier; (iii) synthesis of negatively charged membrane-derived oligosaccharides (MDOs) in the periplasm [50]; (iv) the cytoplasmic membrane as the site of efflux pumps able to extrude SDS; and (v) the cytoplasm itself in the form of the ClpP, ClpX, and ClpB proteases necessary to recycle proteins which have been damaged by the SDS [49]. Thus, SDS resistance in enteric bacteria is a cooperative effort with contributions by five different cellular compartments. We do not expect farnesol resistance in *C. albicans* to be as complicated but the present study is the first step in finding out how farnesol resistance is achieved. Switching from UQ7 to UQ9 is a likely adaptation made by *C. albicans* and *C. dubliniensis* to allow farnesol production.

## Figures and Tables

**Table 1:** Strains used in this study.

Yeast Strains	Genotype Description	Source	UQ
*C. albicans* strains			
SC5314	Wild type (clinical isolate)	[29]	UQ9
WO-1	MTLɑ frequent white/opaque switching	[30]	UQ9
*S. cerevisiae* strains			
BY4741 parent	*BY4741 MATa his3Δ1 leu2Δ0 met15Δ0 ura3Δ0 rho^+^*	[21]	UQ6
*∆coq1*::pYES	*coq1* knockout transformed with empty vector	[21]	none
*∆coq1*::pYES + *At2g34630*	*coq1* knockout transformed with Arabidopsis *At2g34630*	[21]	UQ9
*∆coq1*::pYES + *COQ1*	*coq1* knockout transformed native *COQ1* gene	[21]	UQ6
